# Effect of core strengthening exercises on pain and quality of life in females with primary dysmenorrhea: a scoping review

**DOI:** 10.1186/s12887-026-07117-6

**Published:** 2026-06-15

**Authors:** Dr. Sanjivani Nikhil Kamble, S. P. Prajna Prabha, Amisha Sanjay Ghorpade, Omkar Pise, Dr. Tushar J. Palekar

**Affiliations:** 1 Department of Neuro Physiotherapy, Dr. D. Y. Patil College of Physiotherapy, Dr. D. Y. Patil Vidyapeeth, Pune, India; 2 Department of Paediatric Physiotherapy, Dr. D. Y. Patil College of Physiotherapy, Dr. D. Y. Patil Vidyapeeth, Pune, India; 3 Dr. D. Y. Patil College of Physiotherapy, Dr. D. Y. Patil Vidyapeeth, Pune, India

**Keywords:** Primary dysmenorrhea, Core strengthening exercises, Core stability, Lumbopelvic stabilization, Menstrual pain, Physiotherapy, Exercise therapy

## Abstract

**Background:**

Dysmenorrhea is characterized by recurrent lower abdomen or pelvic pain that may radiate to the legs, inner thighs, and lower back. It is divided into two categories: primary dysmenorrhea (PD) and secondary dysmenorrhea (SD). When the ovulatory cycle is established, which usually happens in the first two years following menarche, PD is defined as menstrual discomfort without any visible pelvic disease. Many teenage girls suffer from primary dysmenorrhea, or excruciating period cramps, which can seriously interfere with their everyday activities, social contacts, and attendance at school. By engaging intrinsic musculature that supports spinal load and neuromuscular control, core strengthening aims to improve lumbar stability. Deep stabilizing muscles are isolated and conditioned by core strength training, which enhances functional stability. Strong core muscles are more resilient to everyday biomechanical strains, such as those brought on by menstruation.

**Objective:**

To map and synthesize the existing evidence regarding the effectiveness of core strengthening exercises on pain intensity and quality of life in females with primary dysmenorrhea.

**Eligibility criteria:**

This scoping review included randomized controlled trials, experimental studies, and quasi-experimental studies published in English between 2019 and 2025 that investigated the effects of core strengthening or lumbopelvic stabilization exercises in females with primary dysmenorrhea. Studies primarily focusing on pharmacological management or unrelated physiotherapy modalities were excluded.

**Sources of evidence:**

Electronic databases including PubMed, Scopus, MEDLINE, Google Scholar, and ClinicalKey were searched.

**Charting methods:**

Data were charted using a predefined extraction framework including study design, participant characteristics, intervention details, outcome measures, and key findings.

**Methods:**

A comprehensive literature search was conducted between January 2019 and March 2025 using keywords including “primary dysmenorrhea,” “core strengthening,” “core stability,” “lumbopelvic exercises,” and “exercise therapy” with Boolean operators (“AND,” “OR”). Eleven studies met the inclusion criteria, comprising randomized controlled trials, comparative studies, and quasi-experimental designs, with a total sample size of 445 participants. The most commonly used outcome measures were the Visual Analog Scale (VAS), Numerical Pain Rating Scale (NPRS), WaLIDD scale, and quality of life questionnaires.

**Results:**

Most included studies demonstrated significant reductions in pain intensity following core strengthening interventions lasting between 6 and 12 weeks. Several studies also reported improvements in quality of life, functional ability, sleep quality, and psychological well-being. Comparative studies showed that core strengthening exercises were more effective than stretching exercises alone in reducing dysmenorrhea symptoms. Studies incorporating lumbopelvic stabilization and pressure biofeedback reported enhanced neuromuscular control and superior pain reduction outcomes. Despite generally positive findings, heterogeneity in study design, exercise protocols, outcome measures, and limited long-term follow-up reduced the overall strength of evidence.

**Conclusion:**

The findings of this scoping review suggest that core strengthening exercises may be an effective non-pharmacological intervention for reducing pain intensity and improving quality of life in females with primary dysmenorrhea. Core stabilization programs appear to enhance lumbopelvic stability and functional outcomes while presenting minimal adverse effects. However, further high-quality randomized controlled trials with standardized protocols and long-term follow-up are required to strengthen the current evidence base.

**Supplementary Information:**

The online version contains supplementary material available at 10.1186/s12887-026-07117-6.

## Introduction

Primary dysmenorrhea (PD) is considered one of the most common female gynaecological problems that affect both adolescents and young reproductive females. It can be described as painful cramps that affect the lower abdominal region without any pelvic problem. Pain associated with PD usually comes in the form of spasms or cramps and might spread towards the lower back, pelvis, groins, and thighs. Besides experiencing pelvic pain, females who suffer from PD are likely to experience other symptoms like nausea, vomiting, diarrhea, headache, dizziness, tiredness, insomnia, and impairment of physical activity. This condition usually occurs 6 to 24 months after menarche when the reproductive cycle is established, and the condition happens before or when menses start and lasts up to 8 to 72 h. [1]. Primary dysmenorrhea is regarded as one of the leading public health problems due to its widespread occurrence and substantial influence on quality of life, academic achievement, psychological well-being, and functioning during the daytime. According to epidemiological research, the incidence of primary dysmenorrhea among menstruating women ranges between 50 and 90%. One study based on a meta-analysis among college students found that the estimated prevalence rate was 66.1%, with prevalence rates having increased in recent times among adolescents and young adults. Adolescents are especially susceptible due to the negative effects that dysmenorrhea can have on school attendance, concentration, exercise, and socialization [2]. Notwithstanding the considerable economic burden linked to dysmenorrhea, women have persisted in utilizing pharmacotherapy, which includes the use of NSAIDs that can have side effects when used for a long time. As a result, non-pharmacological treatments and physiotherapy have become increasingly popular in the treatment of pain.

The pathophysiological causes of primary dysmenorrhea include the overproduction of prostaglandins within the uterus, especially prostaglandin F2α, during menstruation. The high concentration of prostaglandins leads to the intensification of uterine contractions, vasospasm, decreased uterine circulation, and ischemia, causing pain and systemic reactions. Apart from peripheral physiologic causes, there could be neuromuscular dysfunction and central sensitization that could enhance pain sensitivity among women with dysmenorrhea. Considering that dysmenorrhea results from physiologic and musculoskeletal etiologies, exercise therapy has gained popularity in conservative treatments [3].

Physiotherapy treatments based on exercise have been found to be effective in lowering pain and enhancing the quality of life of women suffering from primary dysmenorrhea. Exercise has been hypothesized to alleviate menstrual pain via various methods, including increasing blood flow in the pelvis, releasing endorphins, relaxing muscles, increasing flexibility, regulating pain transmission, and decreasing sympathetic nerve activation. Several forms of exercise like aerobic exercise, stretching exercises, Pilates, lumbopelvic stability, and functional training have been examined in recent times.

Elbandrawy et al. (2021) conducted research on the effects of aerobic and isometric exercise in female subjects suffering from primary dysmenorrhea and concluded that there was significant improvement in terms of reduction of pain and distress after exercise therapy [2]. Berde et al. (2019) conducted another study that showed how chair aerobic exercises and core strengthening exercises successfully relieved the symptoms of dysmenorrhea but core strengthening exercises resulted in relatively more improvements in terms of pain relief and physical performance [4]. A preliminary randomized control trial conducted by Song (2023) found that Pilates exercises had positive impacts on the level of pain, physical function, sleep, and mental state of young dysmenorrheic women [5].

From the various physiotherapy techniques used, core strengthening exercises have become particularly significant within the field due to their impact on enhancing lumbopelvic stability and neuromuscular control. Core muscles refer to the transversus abdominis, multifidus, pelvic floor muscles, diaphragm, and deep muscle stabilizers. These muscles work together to create spinal stability, posture control, load transmission, and body coordination. Inability to activate these muscles may lead to greater mechanical stress in the lumbopelvic joint area and may cause pain and muscle dysfunction in the case of menstrual pain.

The possible ways by which core strength training is effective for women suffering from primary dysmenorrhea include many physiological and biomechanical approaches. Enhanced muscle recruitment of the muscles that stabilize the pelvis can lead to stabilization of the pelvis, decreased muscle fatigue, improved circulation, and lower pain threshold. The increased secretion of endorphins due to exercise can also provide additional pain relief and mental relaxation.

Research specifically conducted on the impact of core strength training has yielded positive results. Specifically, Agrawal et al. (2021), in comparing stretching exercises and core strengthening exercises for young sedentary females suffering from primary dysmenorrhea, concluded that while both types of exercises provided a decrease in menstrual pain, core strengthening exercises performed better [6]. In similar research, Rakholiya et al. (2021) [7] found more efficient pain relief from strengthening exercises than from stretching exercises in adolescent girls with dysmenorrhea. Bağci Çelik et al. (2024) [8] showed that core strength exercises were effective at improving pain intensity and quality of life in adolescents with primary dysmenorrhea. Additionally, according to Şener et al. (2026), [9] lumbopelvic stability exercises combined with pressure biofeedback yielded better results than either approach separately in terms of pain management and neuromuscular control. Shinde (2023) [3] stated that combination exercises were more effective than core strengthening exercises alone.

Despite the support that exists for the efficacy of exercise therapy in managing dysmenorrhea from past studies, the extant literature is still heterogenous with regard to study designs, exercise regimens, outcomes measures used, and methodological quality. Several literature reviews have considered physical therapy intervention strategies, yet few of them have synthesized the evidence for the core strengthening exercises. Further, there has been little literature on the use of lumbo-pelvic stabilization and core strengthening exercises among young girls with primary dysmenorrhea.

Hence, there is a need for the systematic identification of the evidence base for the use of core strengthening exercises in minimizing the intensity of pain and enhancing the quality of life among female patients with primary dysmenorrhea. A scoping review of the literature will assist in achieving this purpose.

## Methods

### Study design

This study was carried out through a scoping review to fully identify and analyze the existing evidence on the efficacy of core strengthening exercises among females with primary dysmenorrhea. The methodology of the study was based on the guidelines provided by the Preferred Reporting Items for Systematic Reviews and Meta-Analyses Extension for Scoping Reviews (PRISMA-ScR). The scoping review approach was used in accordance with the methodological framework of Arksey and O'Malley and further improved by Levac et al. The methodological framework involves the following steps:

- Defining the research question
- Searching for relevant literature
- Screening the articles
- Data charting
- Summarizing the data

Since the aim of the study was not to assess the efficacy of core strengthening exercise in primary dysmenorrhea but rather map the scope and characteristics of the existing evidence, a scoping review approach was chosen.

## Research question

The research question that guided this review was:

"What is the existing evidence on the efficacy of core strengthening exercises in relieving pain and improving quality of life in females with primary dysmenorrhea?"

### Protocol and registration

This scoping review followed the PRISMA-ScR reporting guidelines. Although a formal protocol was not registered prior to commencement of the review, the methodology was predefined by the review team before data collection and screening were initiated. The absence of protocol registration is acknowledged as a limitation of the study.

### Eligibility criteria

Studies were included or excluded on the basis of pre-specified inclusion and exclusion criteria.

#### Inclusion criteria

- Randomized Controlled Trials (RCTs)
- Experimental studies
- Quasi-experimental studies
- Studies that examined core strengthening/core stability/lumbopelvic stabilization exercises in females suffering from primary dysmenorrhea
- Articles written in English language and published between 2019 to 2025
- Studies that assessed pain severity, quality of life, functional impairment or menstrual symptoms

#### Exclusion criteria

- Studies involving secondary dysmenorrhea
- Studies that evaluated medications
- Case reports, abstracts, editorial or letters to editors
- Systematic reviews, narrative reviews and meta-analyses
- Studies not available in full text form
- Studies irrelevant to exercise intervention for primary dysmenorrhea

### Evidence sources

A thorough literature search was performed using the following database sources: PubMed, Scopus, MEDLINE, Google Scholar, and ClinicalKey. The literature review included articles published from January 2019 up to March 2025 to ensure incorporation of the latest evidence regarding exercise-based physiotherapy for the treatment of primary dysmenorrhea.

All the studies considered for inclusion were those written in the English language. The reason behind this limitation is that non-English papers would create translational difficulties.

### Search strategy

The search strategy was formulated by combining keywords, MeSH terms, and Boolean operators. Search terms used were:

- "Primary dysmenorrhea"
- "Menstrual pain"
- "Core strengthening exercises"
- "Core stability"
- "Lumbopelvic stabilization"
- "Exercise therapy"
- "Physiotherapy"
- "Adolescent females"

Boolean operators "AND" and "OR" were employed in the appropriate combination of the search terms. An example of the search strategy on PubMed database is shown below:

("Primary Dysmenorrhea" OR "Menstrual Pain") AND ("Core Strengthening" OR "Core Stability" OR "Lumbopelvic Stabilization") AND ("Exercise Therapy" OR "Physiotherapy").

### Study selection

*T*he study selection procedure comprised two phases. In the first phase, titles and abstracts of studies were screened independently by two reviewers in accordance with the specified eligibility criteria. The second phase entailed conducting full text screening of potential articles.

Any discrepancies between the reviewers at any point of title and abstract screening or full-text screening phase were discussed and resolved. Redundant articles found in multiple databases were eliminated using reference management software.

The process of study selection was reported as per the PRISMA-ScR flow diagram.

### Data charting process

Data charting in this review was guided by a standard data extraction form prepared by the review team. Before the actual data extraction process, this form was tested on a subset of studies to refine its applicability.

Variables extracted from each study included:

- Name and year of publication
- Design of study
- Characteristics of population studied and their age groups
- Sample size
- Intervention type and duration
- Outcome measures employed
- Results of study and conclusion
- Limitations of the study

The data extraction process was performed by one reviewer, while a second reviewer checked for consistency. In case of any inconsistencies, they were discussed and sorted out.

### Critical appraisal

There was no need for critical appraisal in this scoping review since it is not part of the scoping review protocol. This is because scoping reviews do not evaluate the methodological quality of individual studies nor assess interventions but focus more on the nature and range of evidence available.

### Data analysis and presentation

Extracted data were summarized descriptively and presented in tabular format according to the objectives of the review. Studies were categorized based on intervention type, study design, and outcome measures. Narrative synthesis was used to summarize findings related to the effectiveness of core strengthening exercises on pain intensity, functional outcomes, and quality of life in females with primary dysmenorrhea.

## Results

### Study selection and study characteristics

There were 11 studies that were eligible and thus selected in this scoping review. These studies were published within the years 2019 and 2026 and were focused on the exercise-based physiotherapy interventions among females with primary dysmenorrhea. The process of study selection was carried out in accordance with the PRISMA-ScR guidelines.

The included studies had the following characteristics:

- 2 randomized controlled trial studies
- 5 comparative experimental studies
- 3 quasi-experimental studies
- 1 non-randomized control study

All the studies included examined the effects of core strengthening exercise either independently or alongside other exercise interventions among females with primary dysmenorrhea.

### Participants

In the selected studies, the overall sample size was made up of about 465 females suffering from primary dysmenorrhea. Most of the subjects were teenagers and young women because most of the studies used females aged between 13 and 30 years.

Most of the studies focused on adolescent girls and young sedentary women since primary dysmenorrhea is very common among them and has a great impact on their school attendance and physical activities.

### Intervention

The types of interventions discussed in this literature review can be grouped into three main categories:

#### Core strengthening and lumbopelvic stabilization exercises

Interventions associated with core strengthening exercises were the most popular type used in the majority of studies. Core strengthening interventions aimed at activation and strengthening of deep stabilizing muscles like transversus abdominis, multifidus, pelvic floor muscles, and trunk stabilizers.

Agrawal et al. (2021), Rakholiya et al. (2021), Bağci Çelik et al. (2024), Şener et al. (2026), Zainab et al. (2023), and Shinde (2023) assessed core strengthening or lumbopelvic stabilization exercises.

In addition, Şener et al. (2026) applied pressure biofeedback in lumbopelvic stabilization exercises for better neuromuscular control and accuracy of muscle activation.

#### Combination of exercises

Some researchers studied the combination of core strengthening exercises with stretching exercises or used both interventions.

Shinde (2023) studied the effect of combined core strengthening and stretching exercises, whereas Rakholiya et al. (2021) and Agrawal et al. (2021) compared stretching exercises with core strengthening interventions.

#### Other physiotherapy exercise interventions

Other exercise interventions that were studied included aerobic exercises, chair aerobic exercises, Pilates, and functional exercises.

On the other hand, Berde et al. (2019) have compared chair aerobic exercises to core strengthening exercises, while Elbandrawy et al. (2021) have compared aerobic and isometric exercise interventions. Song (2023) studied Pilates exercise training intervention, and Al-Emary (2025) studied functional exercise interventions.

### Outcome measures

The outcome measure that was assessed most commonly among the selected studies was pain intensity. The common outcome measures include the following:

- Visual Analog Scale (VAS)
- Numeric Pain Rating Scale (NPRS)
- WaLIDD scale
- Menstrual Symptom Questionnaire
- Questionnaires for quality of life
- Pittsburgh Sleep Quality Index (PSQI)
- Functional assessments

Some studies have measured psychological measures, sleep quality, physical functions, and limitations of daily activities.

### Synthesis of results

#### Intensity of pain

In all included articles, there was evidence of pain intensity reduction as a result of an exercise-based physiotherapy intervention. Out of the 11 articles included in this review, nine articles indicated that core strengthening or lumbopelvic stabilizations significantly reduced menstrual pain.

Agrawal et al. (2021) indicated that core strength training was more effective in reducing pain severity than stretching exercises among young sedentary females with primary dysmenorrhea. In addition, Rakholiya et al. (2021) indicated superiority of pain reduction with core muscle strengthening as opposed to stretching exercises.

Bağci Çelik et al. (2024) indicated significant improvements in pain intensity and quality of life with core exercise interventions among adolescent females. Zainab et al. (2023) also indicated pain intensity reductions with phase-specific core strengthening exercise program.

Şener et al. (2026) showed that core exercise and pressure biofeedback improved pain reduction and neuromuscular control better than exercise without biofeedback.

#### Comparison with other exercise interventions

Comparative studies between core strengthening exercises and other exercise interventions indicated that core stability interventions were more effective.

For instance, Berde et al. (2019) revealed that both chair aerobic exercise and core strengthening exercise reduced dysmenorrheic symptoms but with higher improvements with core strengthening interventions.

Elbandrawy et al. (2021) showed the positive impacts of aerobic and isometric exercises with higher effectiveness of the former in reducing the menstrual symptoms.

Shinde (2023) proved that stretching and core strengthening exercises were more effective than only core strengthening exercises, which implies the importance of flexibility training for patients with dysmenorrhea.

#### Quality of life and functional results

Some researchers noted the improvement of quality of life, sleep quality, psychological status, and functional results in patients undergoing exercise programs.

Song (2023) revealed that Pilates exercises were efficient in alleviating pain intensity, improving sleep quality, physical functioning, anxiety, and depression in young women with dysmenorrhea.

Al-Emary (2025) found significant improvement in quality of life and functional results after functional exercises.

#### General trends in the studies reviewed

The results from all the selected studies indicated that exercise therapy programs for the treatment of primary dysmenorrhea in females were effective in terms of pain reduction and improvement in the quality of life among patients.

Core strengthening interventions had effects such as:

- Increased lumbo-pelvic stability
- Decreased menstrual pain intensity
- Improved neuromuscular control
- Improved physical function
- Quality of life improvement

However, differences in the type of exercise, intervention period, outcome measurements, and study designs made it difficult to compare the findings from different studies. The reviewed studies were characterized by small sample sizes and short intervention periods of 4 to 12 weeks (Table 1).

**Table 1:** Characteristics and outcomes of studies investigating exercise-based physiotherapy interventions for primary dysmenorrhea

Author and Year	Study Type	Population with Age Group	Outcome Measures	Sample Size	Results	Limitations
Agrawal et al. (2021) [6]– *A Comparative Study of Stretching Exercises Versus Core Strengthening Exercises on Primary Dysmenorrhea in Young Sedentary Females* [6]	Comparative experimental study	Young sedentary females with primary dysmenorrhea; age group: 18–25 years	Visual Analog Scale (VAS), Menstrual Symptom Questionnaire, pain intensity assessment	60 participants	Both stretching and core strengthening exercises significantly reduced pain intensity and menstrual symptoms. Core strengthening exercises demonstrated comparatively greater effectiveness in improving pelvic stability and reducing dysmenorrheic pain	Small sample size, short intervention duration, absence of long-term follow-up, limited generalizability to active females
Bağci Çelik et al. (2024) [8]– *The Effect of Core Exercises on Pain and Quality of Life in Teenagers with Primary Dysmenorrhea Complaints*	Non-randomized controlled study	Teenagers with primary dysmenorrhea; age group: 13–19 years	VAS, Short Form Health Survey (SF-36), Quality of Life scales	52 participants	Core exercises significantly reduced menstrual pain and improved quality of life, particularly physical functioning and emotional well-being	Non-randomized design, possible selection bias, short-term intervention, lack of blinding
Berde et al. (2019) [10]– *Effect of Core Strengthening Exercises and Chair Aerobic Exercises in Primary Dysmenorrhoea*	Experimental comparative study	Females with primary dysmenorrhea; age group: 18–25 years	Numerical Pain Rating Scale (NPRS), Menstrual Distress Questionnaire	50 participants	Both interventions effectively reduced dysmenorrheic symptoms; however, core strengthening exercises showed superior reduction in pain intensity and improved daily functioning	Lack of long-term follow-up and inadequate reporting of exercise adherence
Elbandrawy et al. (2021) [2] – *Comparison between the Effects of Aerobic and Isometric Exercises on Primary Dysmenorrhea*	Comparative intervention study	Young females diagnosed with primary dysmenorrhea; age group: 18–24 years	VAS, Menstrual Distress Questionnaire (MDQ)	105 participants	Aerobic and isometric exercises both significantly reduced pain severity, though aerobic exercise produced greater improvements in overall menstrual distress	Small sample size, short intervention duration, no assessment of psychosocial variables
Rakholiya et al. (2021) [7]– *To Compare the Effect of Stretching and Strengthening Exercises of Core Muscles on Primary Dysmenorrhea in Adolescent Girls*	Comparative study	Adolescent girls with primary dysmenorrhea; age group: 13–19 years	VAS, pain severity scale, activity limitation assessment	100 participants	Core strengthening exercises demonstrated greater efficacy compared to stretching exercises in reducing pain and improving functional activity levels	Absence of randomization, small sample size, short treatment period
Şener et al. (2026) [9]– *Effects of Lumbopelvic and Core Strengthening Exercises with and without Pressure Biofeedback on Primary Dysmenorrhea*	Experimental controlled study	Females with primary dysmenorrhea; age group: 18–30 years	VAS, Quality of Life questionnaires, pressure biofeedback assessment	58 participants	Lumbopelvic and core strengthening exercises significantly reduced pain. Addition of pressure biofeedback improved neuromuscular control and enhanced therapeutic outcomes	Limited availability of long-term evidence and need for multicenter validation studies
Shinde (2023) [3]– *Comparison of Effects of Stretching Exercises and Core Strengthening Exercises Versus Core Strengthening Exercises in Adolescent Girls with Primary Dysmenorrhoea*	Comparative intervention study	Adolescent girls with primary dysmenorrhea; age group: 14–19 years	VAS, Menstrual Symptom Scale	60 participants	Combined stretching and core strengthening exercises were more effective than core strengthening alone in reducing menstrual pain and discomfort	Lack of control group, short duration of intervention, limited external validity
Song (2023) [5]– *Effects of Pilates on Pain, Physical Function, Sleep Quality, and Psychological Factors in Young Women with Dysmenorrhea: A Preliminary Randomized Controlled Study*	Preliminary randomized controlled trial	Young women with dysmenorrhea; age group: 18–30 years	VAS, Pittsburgh Sleep Quality Index (PSQI), depression and anxiety scales, physical function assessment	30 participants	Pilates exercise significantly improved pain intensity, sleep quality, physical functioning, and psychological health parameters	Preliminary study with small sample size and short intervention duration
Zainab et al. (2023) [11]– *A Study to Compare the Effectiveness of Core Strengthening Exercises for Phase I and Phase II of Menstrual Cycle in Primary Dysmenorrhea Subjects*	Comparative experimental study	Females with primary dysmenorrhea; age group: 18–25 years	VAS, menstrual symptom severity assessment	150 participants	Core strengthening exercises during both phases of the menstrual cycle were beneficial, with slightly greater improvement observed during Phase II	Lack of hormonal assessment, limited follow-up, small sample size
Arshiya (2023) [12]– *Immediate Effect of Physiotherapy Intervention in Subjects with Primary Dysmenorrhea: A Quasi-Experimental Study*	Quasi-experimental study	Young females with primary dysmenorrhea; age group: 18–25 years	NPRS, immediate pain relief assessment	26 participants	Physiotherapy interventions produced immediate reduction in pain intensity and muscular discomfort associated with dysmenorrhea	Only immediate effects were assessed; absence of long-term outcome analysis
Al-Emary (2025) [13]– *Effect of Functional Exercises on Pain and Quality of Life in Females with Primary Dysmenorrhea*	Experimental study	Females with primary dysmenorrhea; age group: 18–30 years	VAS, SF-36 Quality of Life Questionnaire	58 participants	Functional exercises significantly reduced pain severity and improved quality of life, especially physical and social functioning domains	Limited methodological details and lack of comparison with other physiotherapy interventions

## Discussion

Dysmenorrhea is one of the most common gynecological conditions among teenage and young women that is associated with painful cramping during menstruation without pelvic disease. Dysmenorrhea causes negative effects on various aspects such as academics, physical functioning, psychological status, sleep disturbance, and quality of life. Over the past few years, there has been an increasing interest in physiotherapy treatments for dysmenorrhea, specifically involving exercise therapy programs. This review focuses on a critical evaluation of prior research concerning stretching exercises, core strengthening exercises, aerobic exercises, Pilates, lumbopelvic stability exercises, and functional exercise interventions for females with primary dysmenorrhea.

Based on the findings from this review, it can be concluded that exercise therapy is very important for pain reduction as well as positive functional outcomes. In terms of interventions, core-strengthening exercises seem to consistently have a positive effect in terms of pain relief as well as improving quality-of-life measures. Agrawal et al. (2021) performed a comparison study on the effect of core-strengthening versus stretching exercises on young, sedentary females with primary dysmenorrhea. It was observed that both interventions effectively reduced dysmenorrheic pain; however, core strengthening exercises proved to be more effective in alleviating pain compared to stretching exercises. The reason for better efficacy in the case of core-strengthening exercises may be attributed to better muscle recruitment, improved blood flow, and reduced uterine ischemia [6].

The same results were obtained by Rakholiya et al. (2021), when comparing stretching exercises to strengthening exercises for core muscles in teenage girls experiencing primary dysmenorrhea. The study found out that strengthening exercises for the core muscles helped achieve higher levels of pain relief than the stretching exercise alone. The mechanism postulated was the enhancement of pelvic stability and neuromuscular control, which help reduce the level of strain put on the pelvis by dysmenorrhea. These results contribute to the accumulating evidence that stabilization exercises for abdominal, pelvic, and lumbopelvic muscles can be helpful in managing dysmenorrhea [7].

Supporting the use of core stabilization exercises was Bağci Çelik et al. (2024), whose study examined the impact of core exercise intervention on the level of pain and quality of life in teenage females suffering from primary dysmenorrhea. It was discovered that there was a significant decrease in the level of pain experienced and positive changes in the areas related to the quality of life after the implementation of core exercises. These exercises did not only contribute to better physical performance but also improved the subject's emotional and social state. As mentioned, engagement in such exercises regularly can help in endorphin release and increase circulation and relaxation of muscles around the pelvis [8].

Berde et al. (2019) conducted a study on the efficacy of core strengthening exercises and chair aerobic exercises for treating primary dysmenorrhea in women. While both treatment modalities proved successful in alleviating pain and other associated menstrual issues, the core strengthening exercise group demonstrated better results in terms of pain relief and overall improvement in everyday activities. Core strengthening exercises may have a direct effect on pelvic alignment, spine stability, and abdominal muscle endurance, hence reducing pain sensitivity. Chair aerobic exercises are also useful since aerobic exercises improve blood flow and encourage endorphin release, producing an analgesic effect.

The efficacy of aerobic exercise as a complementary strategy in dysmenorrhea management was examined further by Elbandrawy et al. (2021). The researchers conducted research on females with primary dysmenorrhea and compared the effect of aerobic exercises to that of isometric exercises. The outcome of their study revealed that both forms of exercise were efficacious in relieving pain and discomfort associated with menstruation, but aerobic exercise had a more pronounced effect on alleviating the discomfort caused by menstruation. Exercise in general promotes oxygenation of body tissues, reduces sympathetic nervous system activity, and increases the secretion of endorphins.

There is evidence that the combined approach of stretching and strength training exercises has yielded positive outcomes as well. In a study conducted by Shinde (2023), the effects of stretching and core strength exercises were compared with those of core strength exercises alone among adolescent girls with primary dysmenorrhea. The findings of the study revealed that the group that underwent combined treatment exhibited better improvement in pain intensity and menstrual symptoms than the group treated only with core strength exercises. The researchers suggested that while stretching exercises help enhance muscle flexibility and prevent muscle tightness in the pelvic area, strength training increases stabilization and endurance [3].

The latest developments in the field of lumbopelvic rehabilitation include the incorporation of pressure biofeedback in exercises. Şener et al. (2026) examined the impact of performing lumbopelvic and core strengthening exercises either with or without pressure biofeedback among women suffering from primary dysmenorrhea. Their findings revealed that both methods were effective in reducing pain; yet, subjects who performed exercises in combination with pressure biofeedback showed better outcomes concerning their reduced pain levels and quality of life. The scientists argued that biofeedback helps to activate and control the activity of deep muscles like the transversus abdominis and the pelvic floor muscles [9].

Other than traditional muscle strengthening exercises, there are mind–body exercise programs that have been found to be effective. In particular, Pilates, which focuses on controlled breathing, core activation, posture correction, flexibility, and muscular endurance, has proven its efficacy. According to Song (2023), Pilates significantly reduced menstrual pain, improved sleep quality, physical function, anxiety, and depression among young females suffering from dysmenorrhea in a preliminary randomized controlled trial. The improvement in physical function, sleep quality, and neuromuscular coordination and autonomic regulation may result from increased core activation and postural correction. The reduction in anxiety and depression among patients with dysmenorrhea is important because dysmenorrhea often results in emotional upset and irritability [5].

In addition, studies have also been done on the appropriate time for carrying out exercise interventions in relation to the menstrual cycle. In their study, Zainab et al. (2023) conducted research to compare the effect of core strengthening exercises carried out in Phase I and Phase II of the menstrual cycle in females with primary dysmenorrhea. The findings indicated that there was an improvement in the level of pain among participants who undertook exercise in either phase, but those in Phase II showed more improvement than the rest [11].

Immediate interventions through physiotherapy have also been seen to have some positive effects. According to Arshiya (2023), a quasi-experimental study was conducted to determine the immediate impact of physiotherapy interventions in patients with primary dysmenorrhea. The findings revealed that there was an immediate decrease in the severity of pain and muscle discomfort after the intervention. This is evidence that physiotherapy can be used as an effective non-pharmacological method for offering quick relief from symptoms. However, the study did not consider the long-term impacts of the interventions [12].

In recent times, (18)Al-Emary (2025) conducted research to analyze the impact of functional exercises on the pain level and quality of life in female patients suffering from primary dysmenorrhea. Functional exercise interventions have been found to reduce pain intensity as well as improve physical and psychosocial aspects of quality of life. Functional exercises simulate real-life activities and contribute to enhancing muscle coordination, stamina, and balance. The results of the study show that functional exercises can offer significant benefits for women suffering from dysmenorrhea [13].

Together, the above-stated research findings prove the efficacy of physiotherapy treatments for the treatment of primary dysmenorrhea. The use of core strengthening exercises has repeatedly proven to be an effective approach to pain relief and functional improvement. Additionally, stretching exercises, aerobic exercises, Pilates, functional exercises, and lumbopelvic stability exercises have shown positive therapeutic effects on patients with primary dysmenorrhea. The possible physiological basis of such positive results could include better blood supply to the pelvis, secretion of endorphins, decreased muscle tension, improved neuromuscular control, and lumbopelvic stability.

In spite of all these positive results, there are some limitations that need to be mentioned regarding the methods used in these studies. First, most of these studies used rather small samples; thus, generalizations cannot be made easily. Second, many treatments lasted for a short period of time without any follow-ups; hence, it is hard to see how sustainable the effects of treatment will be. Third, in many studies, the use of randomization was not applied; thus, it is harder to prove that there was no bias in their findings.

In summary, the existing literature shows that physiotherapy exercise protocols are effective in alleviating pain and enhancing quality of life in women with primary dysmenorrhea. Of these exercises, core strengthening seems especially useful, given its benefits in terms of lumbo-pelvic stabilization, neuromuscular control, and pain modulation. Adding stretching, aerobic exercise, Pilates, and functional training to the physiotherapy protocol can improve treatment results even more. Thus, physiotherapy should be viewed as an important aspect of conservative management of primary dysmenorrhea.

However, the scoping review revealed that there is a lack of knowledge regarding the positive impact of physiotherapy for menstrual cramps. The impact of physiotherapy on the health status of adolescents remains under investigation, which explains this limitation. Women's health problems, including menstrual pain, have not been studied adequately, resulting in the lack of sufficient evidence. Physical therapy is gaining popularity since it is a safe and simple approach to address certain issues faced by women and adolescents. Additional research on physical exercise for dysmenorrhea needs to be conducted.

The current investigation, by virtue of using available data and preparing for future studies, helps contribute to the growing pool of knowledge in the field. Hopefully, gaps present at the moment will be filled by future, more detailed investigations as women’s health grows increasingly important in the context of physiotherapy. Future research in the area of women's health will be helped by advances in science and medicine along with increased public interest in the matter. Previous studies have indicated that therapeutic exercise may aid in reducing dysmenorrhea symptoms. Not much has been researched on this topic, however, and the underlying mechanisms remain unknown. Future studies should focus on determining the best type of exercises, their duration, frequency, and intensity. With its insight into symptom management, this paper will likely be useful in improving the lives of women and teenagers suffering from dysmenorrhea.

### Limitations

There are numerous limitations on the scoping review presented above. It may be that relevant literature has been omitted from consideration owing to the exclusion of articles that were not written in English. Additionally, the lack of quality assessment for the article means that it is impossible to discuss the strength of the available evidence. Comparing studies is also difficult because of different exercise programs, measures used, and methods applied.

## Conclusion

This study found that the most popular form of therapeutic physical activity to reduce menstruation discomfort in the examined studies was core strengthening exercises, especially among women in their 20 s. One effective non-pharmacological treatment for dysmenorrhea may be aerobic exercise.

Numerous workout plans have been examined, showing a variety of approaches in the research that is now available. Exercise helps alleviate menstrual cramps, according to the evaluated studies. However, it is challenging to draw firm conclusions due to the broad diversity of exercise methods, durations, and intensities. Additionally, the dearth of high-caliber studies that methodically assess these regimens' effectiveness highlights the need for greater research.

Future studies should standardize exercise routines, determine the most beneficial types and dosages, and investigate the long-term advantages for dysmenorrhea patients.

To guarantee generalizability and inclusiveness, studies should include women with a range of ages, levels of activity, and menstrual health profiles. This study suggests that therapeutic vigorous physical activity can effectively cure dysmenorrhea symptoms, despite its limitations.

Physiotherapists and other medical professionals can more effectively include exercise therapy into the treatment of women with menstruation discomfort because to the emphasis on ongoing research.

## Supplementary Information

Supplementary Material 1.

## Data Availability

All data analysed during this study are included in this published article. Further details can be made available from the corresponding author upon reasonable request.

## Abbreviations

PD: Primary Dysmenorrhea
SD: Secondary Dysmenorrhea
RCT: Randomized Controlled Trial
VAS: Visual Analogue Scale
NPRS: Numeric Pain Rating Scale
WaLIDD: Working ability, Location, Intensity, Days of pain, Dysmenorrhea score
PedsQL: Pediatric Quality of Life Inventory
VMSS: Verbal Multidimensional Scoring System
MMDQ: Moos Menstrual Distress Questionnaire
HRQOL: Health-Related Quality of Life
RSS: Retrospective Symptom Scale
PRISMA-ScR: Preferred Reporting Items for Systematic Reviews and Meta-Analyses Extension for Scoping Reviews
PT: Physiotherapy
